# Supplementary material for: Neural activities in music frogs reveal call variations and phylogenetic relationships within the genus Nidirana
Source: Commun Biol. 2022 Jun 6;5:550. doi: 10.1038/s42003-022-03504-8 (PMC9170687; doi:10.1038/s42003-022-03504-8)
Supplement: Supplementary file 2 — Supplemental Information [file 42003_2022_3504_MOESM2_ESM.pdf]

Neural activities in music frogs reveal call variations and phylogenetic relationships within the genus *Nidirana*

Journal name: *Communications Biology*

Ke Fang [1, 2, 3], Yezhong Tang[1], Baowei Zhang[2], Guangzhan Fang[1,4] *

[1] Chengdu Institute of Biology, Chinese Academy of Sciences. No.9 Section 4, Renmin Nan Road, 610041, Chengdu, Sichuan, China

[2] School of Life Science, Anhui University, No. 111 Jiulong Road, 230601, Hefei, Anhui, China

[3] Institute of Bio-inspired Structure and Surface Engineering, Nanjing University of Aeronautics and Astronautics, No. 29 Yudao Street, 210016, Nanjing, Jiangsu, China

[4] Key Laboratory of Southwest China Wildlife Resources Conservation (Ministry of Education), China West Normal University, No. 1 Shida Road, 637009, Nanchong, Sichuan, China

* Authors for correspondence: fanggz@cib.ac.cn

Address: No.9 Section 4, Renmin Nan Road, Chengdu, Sichuan, China

Chengdu Institute of Biology, Chinese Academy of Sciences

Post Code: 610041

Tel: +86-28-82890628

Supplementary materials

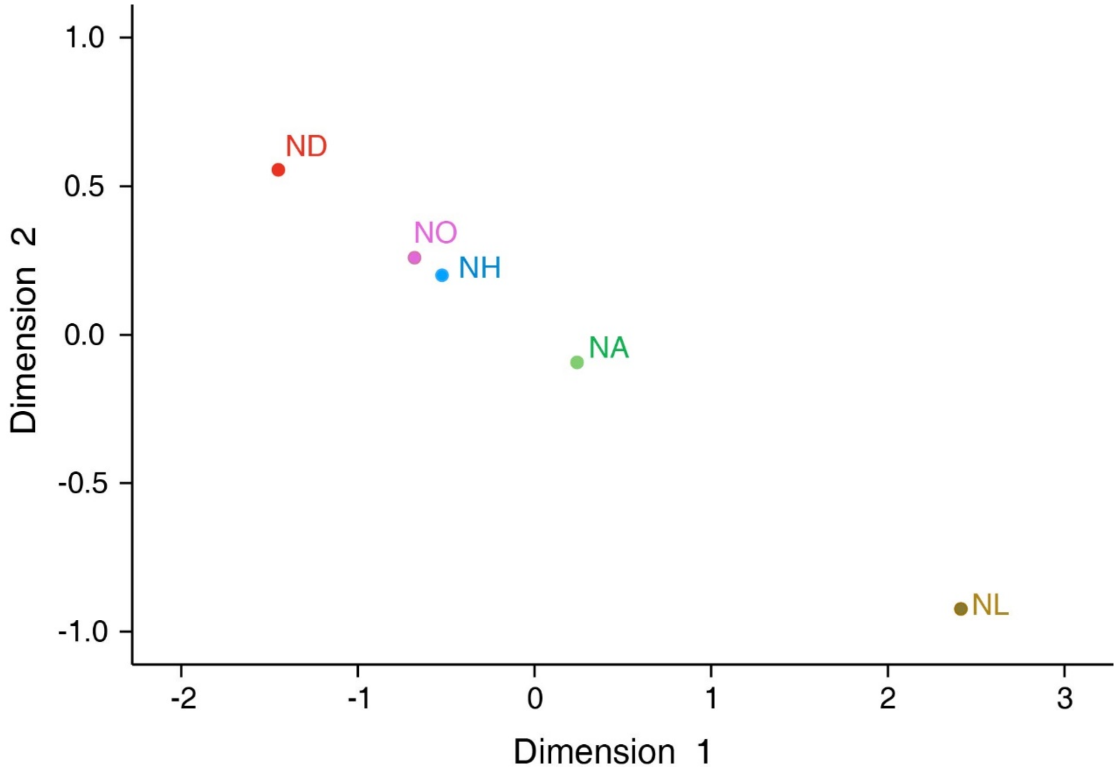

Figure 1. The results of multidimensional scaling analysis. Graphs illustrating the results of two-dimensional multidimensional scaling solutions for the calls of the five *Nidirana* species (n=4 biologically independent samples for each species). Note that mean values of each parameter across the four stimulus exemplars for each species were used for analysis. Abbreviation: ND, the call of *N. daunchina*; NO, the call of *N. okinavana*; NH, the call of *N. hainanensis*; NA, the call of *N. adenopleura*; NL, the call of *N. lini*.

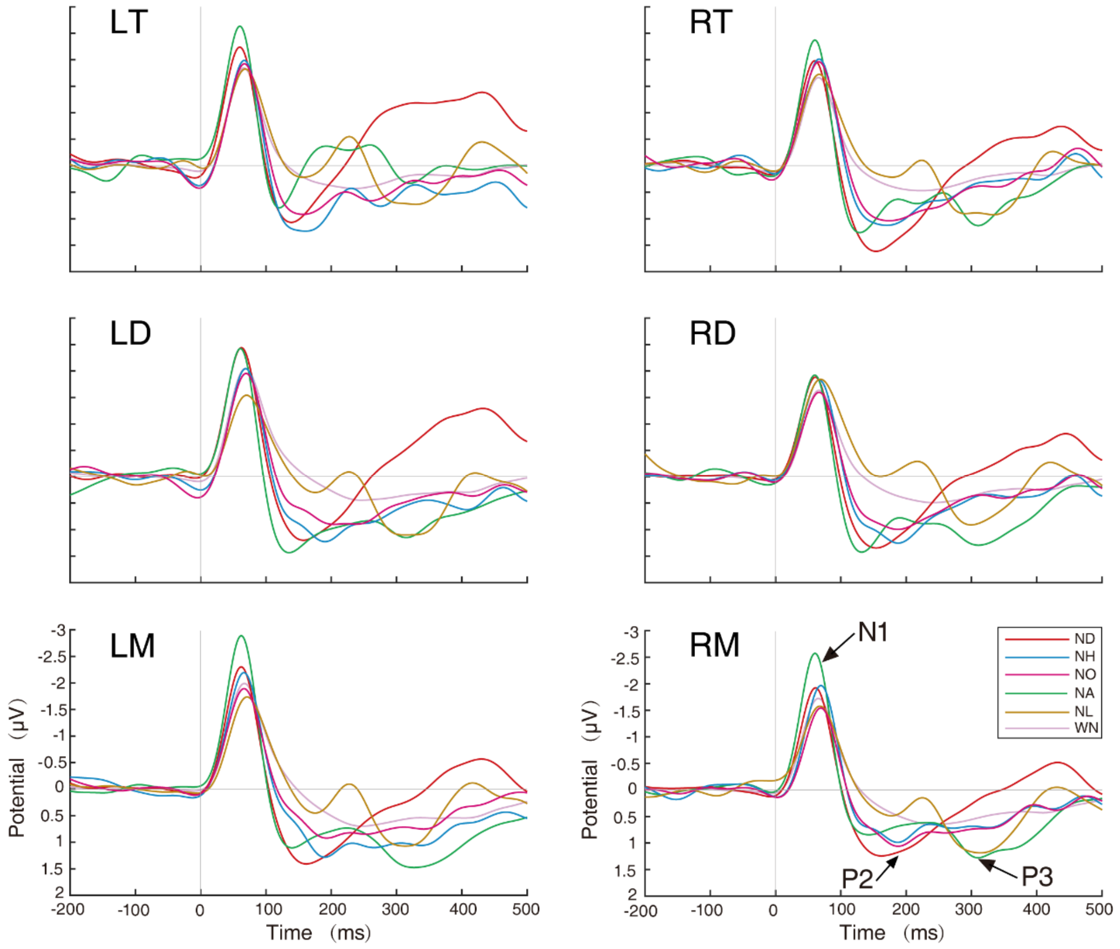

Figure 2. Grand average waveforms for the left and right telencephalon (LT and RT), diencephalon (LD and RD) and mesencephalon (LM and RM) during playbacks of different acoustic stimuli (n=16 biologically independent animals). Abbreviations: ND, the call of *N. daunchina*; NH, the call of *N. hainanensis*; NO, the call of *N. okinavana*; NA, the call of *N. adenopleura*; NL, the call of *N. lini*; WN, white noise.

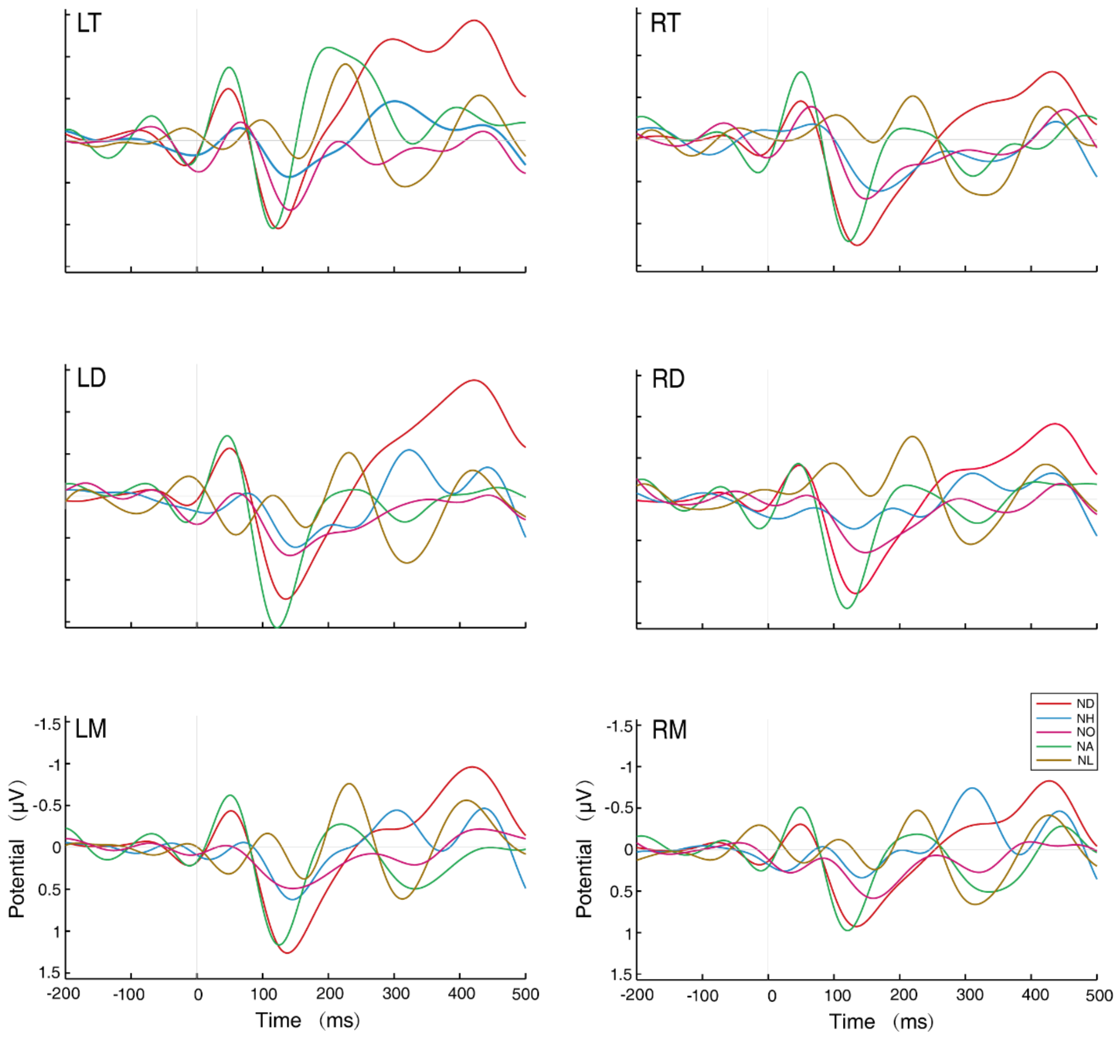

Figure 3. Difference waveforms for the left and right telencephalon (LT and RT), diencephalon (LD and RD) and mesencephalon (LM and RM) during playbacks of different acoustic stimuli (n=16 biologically independent animals). Note that difference waves were acquired by subtracting the ERP amplitudes in response to white noise from that to each species' advertisement calls, which could be used to compare relative variations between the ERP responses to the various calls. Abbreviations: ND, the call of *N. daunchina*; NH, the call of *N. hainanensis*; NO, the call of *N. okinavana*; NA, the call of *N. adenopleura*; NL, the call of *N. lini*.

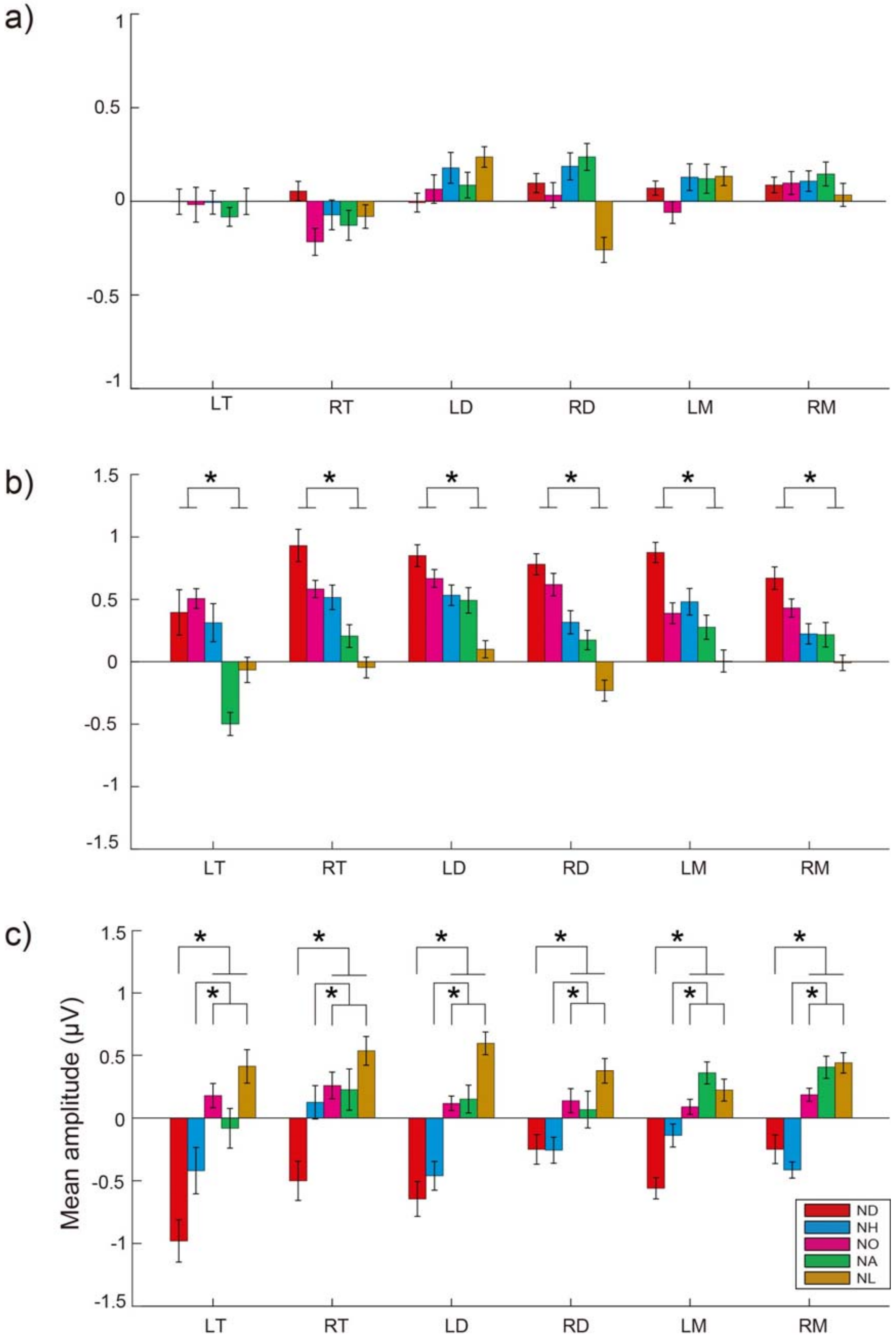

Figure 4. Means and standard errors of N1 (a), P2 (b) and P3a (c) amplitudes for each stimulus and each brain area. Each asterisk indicates that there are significant differences in amplitudes between different acoustic stimuli (n=16 biologically

independent animals; $p < 0.05$; three-way repeated measures ANOVA). Note that for each ERP component the significant differences among brain areas did not shown for a better demonstration. Abbreviation: ND, the call of *N. daunchina*; NH, the call of *N. hainanensis*; NO, the call of *N. okinavana*; NA, the call of *N. adenopleura*; NL, the call of *N. lini*; LT and RT indicate the left and right sides of the telencephalon; LD and RD indicate the left and right sides of the diencephalon; LM and RM indicate the left and right sides of the mesencephalon.

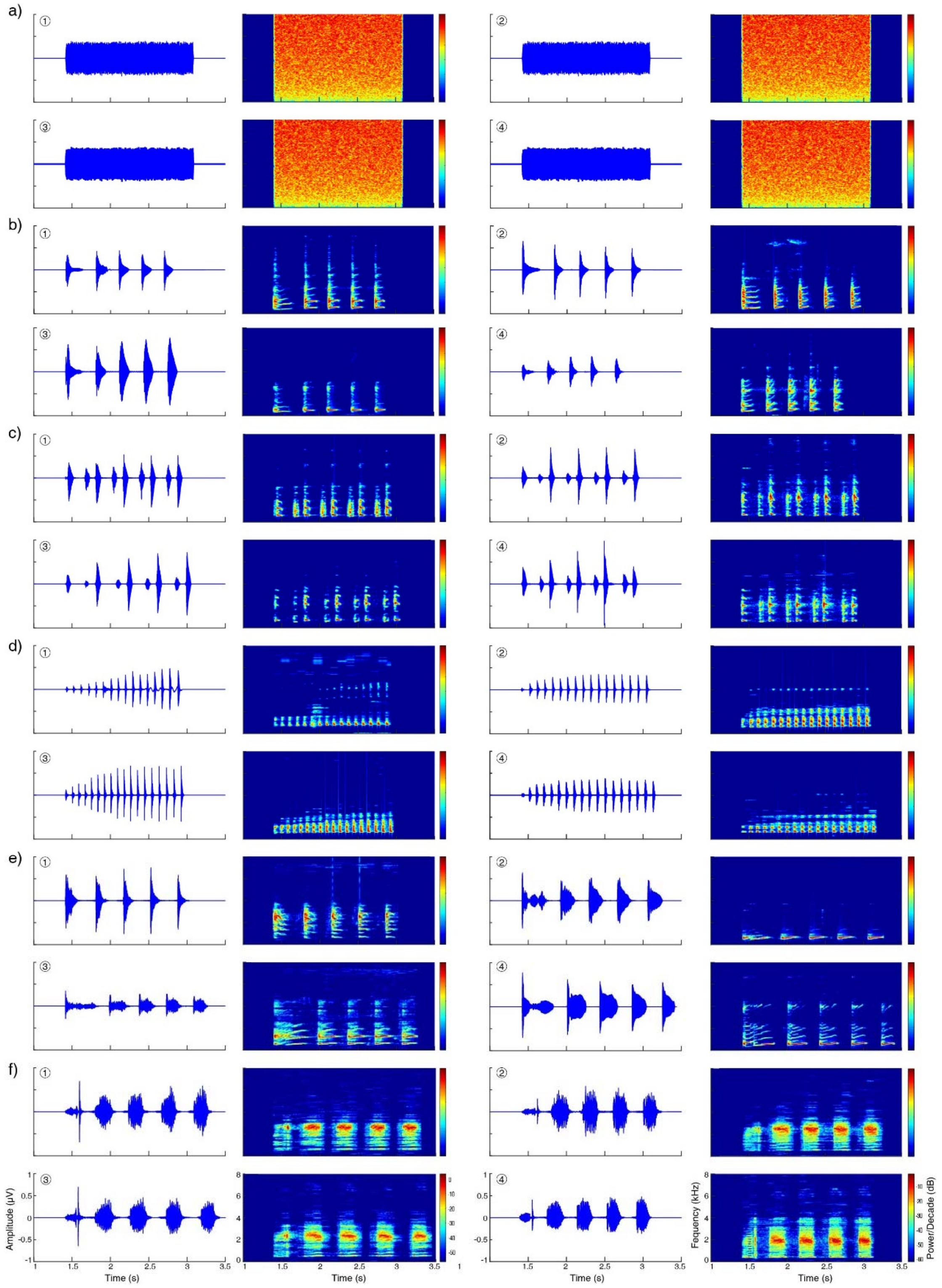

Figure 5. Waveforms and spectrograms of the twenty-four stimuli: a) white noise; **b)** the four calls of *N. daunchina*; **c)** the four calls of *N. hainanensis*; **d)** the four calls of *N. okinavana*; **e)** the four calls of *N. adenopleura*; **f)** the four calls of *N. lini*.

Table 1. The acoustic parameters measured for the four calls of *N. daunchina*

Parameters	Definition	*ND* 1	*ND* 2	*ND* 3	*ND* 4	Mean ± SD
FF1	Fundamental frequency of the 1st note (Hz)	430.600	515.600	421.800	473.700	460.425±37.423
FF2	Fundamental frequency of the 2nd note (Hz)	559.800	609.300	562.500	517.600	562.300±32.455
FF3	Fundamental frequency of the 3rd note (Hz)	602.900	656.200	609.300	559.800	607.050±34.171
FF4	Fundamental frequency of the 4th note (Hz)	602.900	656.200	609.300	559.800	607.050±34.171
FF5	Fundamental frequency of the 5th note (Hz)	603.900	656.200	609.300	559.800	607.300±34.143
DF1	Dominant frequency of the 1st note (Hz)	818.200	515.600	421.800	904.300	664.975±201.371
DF2	Dominant frequency of the 2nd note (Hz)	1119.000	609.300	562.500	517.600	702.100±242.871
DF3	Dominant frequency of the 3rd note (Hz)	602.900	656.200	609.300	559.800	607.050±34.171
DF4	Dominant frequency of the 4th note (Hz)	602.900	656.200	609.300	559.800	607.050±34.171
DF5	Dominant frequency of the 5th note (Hz)	602.900	656.200	609.300	559.800	607.050±34.171
ND1	Duration of the 1st note (sec)	0.235	0.219	0.216	0.170	0.210±0.024
ND2	Duration of the 2nd note (sec)	0.155	0.113	0.131	0.122	0.130±0.016
ND3	Duration of the 3rd note (sec)	0.144	0.118	0.133	0.109	0.126±0.013
ND4	Duration of the 4th note (sec)	0.139	0.111	0.120	0.101	0.118±0.014
ND5	Duration of the 5th note (sec)	0.123	0.110	0.119	0.119	0.118±0.005
RT1	Rise time of the 1st note (sec)	0.024	0.007	0.032	0.020	0.021±0.009
RT2	Rise time of the 2nd note (sec)	0.008	0.007	0.014	0.013	0.011±0.003
RT3	Rise time of the 3rd note (sec)	0.009	0.006	0.028	0.020	0.029±0.019
RT4	Rise time of the 4th note (sec)	0.011	0.007	0.030	0.013	0.015±0.009
RT5	Rise time of the 5th note (sec)	0.008	0.007	0.027	0.018	0.015±0.008
FT1	Fall time of the 1st note (sec)	0.210	0.207	0.167	0.134	0.180±0.031
FT2	Fall time of the 2nd note (sec)	0.143	0.103	0.117	0.101	0.116±0.017
FT3	Fall time of the 3rd note (sec)	0.134	0.104	0.102	0.088	0.107±0.017
FT4	Fall time of the 4th note (sec)	0.124	0.102	0.090	0.074	0.098±0.018

FT5	Fall time of the 5th note (sec)	0.114	0.103	0.091	0.099	0.102±0.008
INI1	Inter-note interval between the 1st and 2nd notes (sec)	0.163	0.160	0.160	0.151	0.159±0.005
INI2	Inter-note interval between the 2nd and 3rd notes (sec)	0.138	0.180	0.155	0.152	0.156±0.015
INI3	Inter-note interval between the 3rd and 4th notes (sec)	0.148	0.189	0.157	0.168	0.166±0.015
INI4	Inter-note interval between the 4th and 5th notes (sec)	0.152	0.200	0.165	0.200	0.179±0.021
CD	Call duration (sec)	1.406	1.420	1.343	1.322	1.373±0.041

Table 2. The acoustic parameters measured for the four calls of *N. hainanensis*

Parameters	Definition	*NH* 1	*NH* 2	*NH* 3	*NH* 4	Mean ± SD
FF1	Fundamental frequency of the 1st note (Hz)	602.900	689.000	689.000	689.000	667.475±37.282
FF2	Fundamental frequency of the 2nd note (Hz)	559.800	645.900	732.100	602.900	635.175±63.704
FF3	Fundamental frequency of the 3rd note (Hz)	517.600	689.000	732.100	602.900	635.400±82.397
FF4	Fundamental frequency of the 4th note (Hz)	559.800	732.100	812.300	732.100	709.075±92.194
FF5	Fundamental frequency of the 5th note (Hz)	559.800	732.100	775.100	732.100	699.775±82.699
DF1	Dominant frequency of the 1st note (Hz)	602.900	689.000	689.000	689.000	667.475±37.282
DF2	Dominant frequency of the 2nd note (Hz)	559.800	645.900	732.100	2153.000	1022.700±655.416
DF3	Dominant frequency of the 3rd note (Hz)	517.600	689.000	732.100	602.900	635.400±82.397
DF4	Dominant frequency of the 4th note (Hz)	559.800	732.100	812.300	1894.000	999.550±524.408
DF5	Dominant frequency of the 5th note (Hz)	559.800	732.100	775.100	2153.000	1055.000±639.030
ND1	Duration of the 1st note (sec)	0.140	0.080	0.080	0.075	0.094±0.027
ND2	Duration of the 2nd note (sec)	0.221	0.225	0.219	0.204	0.217±0.008
ND3	Duration of the 3rd note (sec)	0.233	0.232	0.221	0.211	0.224±0.009
ND4	Duration of the 4th note (sec)	0.221	0.225	0.225	0.217	0.222±0.003
ND5	Duration of the 5th note (sec)	0.218	0.230	0.223	0.210	0.220±0.007
RT1	Rise time of the 1st note (sec)	0.050	0.016	0.035	0.018	0.030±0.014
RT2	Rise time of the 2nd note (sec)	0.162	0.167	0.164	0.138	0.158±0.012
RT3	Rise time of the 3rd note (sec)	0.159	0.162	0.172	0.144	0.159±0.010
RT4	Rise time of the 4th note (sec)	0.160	0.164	0.167	0.146	0.159±0.008
RT5	Rise time of the 5th note (sec)	0.154	0.168	0.164	0.145	0.158±0.009
FT1	Fall time of the 1st note (sec)	0.072	0.061	0.046	0.056	0.059±0.009
FT2	Fall time of the 2nd note (sec)	0.062	0.057	0.056	0.062	0.059±0.003
FT3	Fall time of the 3rd note (sec)	0.058	0.065	0.050	0.059	0.058±0.005
FT4	Fall time of the 4th note (sec)	0.059	0.060	0.055	0.054	0.057±0.003

FT5	Fall time of the 5th note (sec)	0.061	0.063	0.059	0.047	0.058±0.006
INI1	Inter-note interval between the 1st and 2nd notes (sec)	0.110	0.131	0.167	0.155	0.141±0.022
INI2	Inter-note interval between the 2nd and 3rd notes (sec)	0.130	0.138	0.178	0.153	0.150±0.018
INI3	Inter-note interval between the 3rd and 4th notes (sec)	0.130	0.127	0.155	0.143	0.139±0.011
INI4	Inter-note interval between the 4th and 5th notes (sec)	0.130	0.140	0.155	0.173	0.150±0.016
CD	Call duration (sec)	1.533	1.539	1.648	1.521	1.560±0.051

Table 3. The acoustic parameters measured for the four calls of *N. okinavana*

Parameters	Definition	*NO* 1	*NO* 2	*NO* 3	*NO* 4	Mean ± SD
FF1	Fundamental frequency of the 1st note (Hz)	775.100	732.100	602.900	703.100	703.300±63.374
FF2	Fundamental frequency of the 2nd note (Hz)	775.100	732.100	645.900	703.100	714.050±46.950
FF3	Fundamental frequency of the 3rd note (Hz)	775.100	732.100	645.900	750.000	725.775±48.579
FF4	Fundamental frequency of the 4th note (Hz)	775.100	775.100	645.900	750.000	736.525±53.316
FF5	Fundamental frequency of the 5th note (Hz)	775.100	775.100	689.000	750.000	747.300±35.185
DF1	Dominant frequency of the 1st note (Hz)	1291.000	1265.000	1076.000	703.100	1083.775±233.454
DF2	Dominant frequency of the 2nd note (Hz)	775.100	1078.000	645.900	703.100	809.000±191.640
DF3	Dominant frequency of the 3rd note (Hz)	775.100	1125.000	645.900	750.000	840.300±205.750
DF4	Dominant frequency of the 4th note (Hz)	775.100	1125.000	645.900	750.000	840.300±205.750
DF5	Dominant frequency of the 5th note (Hz)	775.100	1171.000	689.000	750.000	870.000±214.291
ND1	Duration of the 1st note (sec)	0.045	0.044	0.040	0.042	0.043±0.002
ND2	Duration of the 2nd note (sec)	0.031	0.028	0.040	0.041	0.035±0.006
ND3	Duration of the 3rd note (sec)	0.032	0.025	0.034	0.038	0.032±0.005
ND4	Duration of the 4th note (sec)	0.023	0.025	0.030	0.037	0.029±0.006
ND5	Duration of the 5th note (sec)	0.022	0.022	0.035	0.033	0.028±0.006
RT1	Rise time of the 1st note (sec)	0.009	0.011	0.008	0.008	0.009±0.001
RT2	Rise time of the 2nd note (sec)	0.007	0.007	0.007	0.013	0.009±0.003
RT3	Rise time of the 3rd note (sec)	0.013	0.007	0.007	0.010	0.009±0.002
RT4	Rise time of the 4th note (sec)	0.004	0.005	0.006	0.008	0.006±0.001
RT5	Rise time of the 5th note (sec)	0.006	0.006	0.004	0.006	0.006±0.001
FT1	Fall time of the 1st note (sec)	0.020	0.030	0.028	0.026	0.026±0.004
FT2	Fall time of the 2nd note (sec)	0.020	0.036	0.027	0.023	0.027±0.006
FT3	Fall time of the 3rd note (sec)	0.019	0.028	0.028	0.027	0.026±0.004
FT4	Fall time of the 4th note (sec)	0.018	0.028	0.028	0.026	0.025±0.004

FT5	Fall time of the 5th note (sec)	0.018	0.024	0.028	0.026	0.024±0.004
INI1	Inter-note interval between the 1st and 2nd notes (sec)	0.051	0.055	0.040	0.050	0.049±0.006
INI2	Inter-note interval between the 2nd and 3rd notes (sec)	0.053	0.045	0.047	0.004	0.037±0.019
INI3	Inter-note interval between the 3rd and 4th notes (sec)	0.068	0.063	0.048	0.050	0.057±0.008
INI4	Inter-note interval between the 4th and 5th notes (sec)	0.058	0.063	0.050	0.053	0.056±0.005
CD	Call duration (sec)	1.530	1.554	1.552	1.615	1.563±0.032

Table 4. The acoustic parameters measured for the four calls of *N. adenopleura*

Parameters	Definition	*NA* 1	*NA* 2	*NA* 3	*NA* 4	Mean ± SD
FF1	Fundamental frequency of the 1st note (Hz)	645.900	559.800	559.800	602.900	592.100±35.699
FF2	Fundamental frequency of the 2nd note (Hz)	689.000	689.000	645.900	602.900	656.700±35.699
FF3	Fundamental frequency of the 3rd note (Hz)	732.100	689.000	645.900	602.900	667.475±48.154
FF4	Fundamental frequency of the 4th note (Hz)	732.100	689.000	645.900	645.900	678.225±35.737
FF5	Fundamental frequency of the 5th note (Hz)	732.100	689.000	645.900	645.900	678.225±35.737
DF1	Dominant frequency of the 1st note (Hz)	2540.000	559.800	1291.000	602.900	1248.425±800.138
DF2	Dominant frequency of the 2nd note (Hz)	2368.000	689.000	1248.000	602.900	1226.975±703.787
DF3	Dominant frequency of the 3rd note (Hz)	2540.000	689.000	1291.000	602.900	1280.725±773.864
DF4	Dominant frequency of the 4th note (Hz)	2540.000	689.000	1335.000	645.900	1302.475±764.848
DF5	Dominant frequency of the 5th note (Hz)	2540.000	689.000	1291.000	645.900	1291.475±764.617
ND1	Duration of the 1st note (sec)	0.165	0.324	0.442	0.510	0.360±0.131
ND2	Duration of the 2nd note (sec)	0.174	0.203	0.283	0.307	0.242±0.055
ND3	Duration of the 3rd note (sec)	0.130	0.197	0.216	0.308	0.213±0.064
ND4	Duration of the 4th note (sec)	0.105	0.204	0.199	0.231	0.185±0.048
ND5	Duration of the 5th note (sec)	0.117	0.194	0.190	0.204	0.176±0.035
RT1	Rise time of the 1st note (sec)	0.013	0.009	0.005	0.007	0.009±0.003
RT2	Rise time of the 2nd note (sec)	0.005	0.009	0.015	0.006	0.009±0.004
RT3	Rise time of the 3rd note (sec)	0.007	0.031	0.010	0.006	0.014±0.010
RT4	Rise time of the 4th note (sec)	0.006	0.031	0.008	0.012	0.014±0.010
RT5	Rise time of the 5th note (sec)	0.007	0.008	0.011	0.010	0.009±0.002
FT1	Fall time of the 1st note (sec)	0.150	0.316	0.414	0.499	0.345±0.130
FT2	Fall time of the 2nd note (sec)	0.136	0.188	0.253	0.299	0.219±0.062
FT3	Fall time of the 3rd note (sec)	0.139	0.160	0.205	0.320	0.206±0.070
FT4	Fall time of the 4th note (sec)	0.109	0.163	0.193	0.225	0.173±0.043

FT5	Fall time of the 5th note (sec)	0.136	0.185	0.178	0.194	0.173±0.022
INI1	Inter-note interval between the 1st and 2nd notes (sec)	0.233	0.180	0.160	0.211	0.196±0.028
INI2	Inter-note interval between the 2nd and 3rd notes (sec)	0.207	0.166	0.107	0.209	0.172±0.041
INI3	Inter-note interval between the 3rd and 4th notes (sec)	0.227	0.175	0.130	0.206	0.185±0.037
INI4	Inter-note interval between the 4th and 5th notes (sec)	0.237	0.192	0.152	0.243	0.206±0.037
CD	Call duration (sec)	1.619	1.836	1.863	2.446	1.941±0.307

Table 5. The acoustic parameters measured for the four calls of *N. lini*

Parameters	Definition	*NL* 1	*NL* 2	*NL* 3	*NL* 4	Mean ± SD
FF1	Fundamental frequency of the 1st note (Hz)	517.600	1378.000	1464.000	430.600	947.550±475.421
FF2	Fundamental frequency of the 2nd note (Hz)	602.900	1335.000	1378.000	473.700	947.400±411.923
FF3	Fundamental frequency of the 3rd note (Hz)	602.900	1421.000	1378.000	430.600	958.125±415.818
FF4	Fundamental frequency of the 4th note (Hz)	645.900	1464.000	1335.000	473.700	979.650±426.686
FF5	Fundamental frequency of the 5th note (Hz)	602.900	1421.000	1335.000	473.700	958.150±423.421
DF1	Dominant frequency of the 1st note (Hz)	2713.000	2540.000	2368.000	430.600	2012.900±921.648
DF2	Dominant frequency of the 2nd note (Hz)	2583.000	2445.000	2454.000	1937.000	2354.750±247.290
DF3	Dominant frequency of the 3rd note (Hz)	2540.000	2454.000	2411.000	1808.000	2303.250±289.680
DF4	Dominant frequency of the 4th note (Hz)	2540.000	2454.000	2368.000	2042.000	2351.000±188.481
DF5	Dominant frequency of the 5th note (Hz)	2540.000	2239.000	2368.000	1894.000	2260.250±236.886
ND1	Duration of the 1st note (sec)	0.224	0.233	0.271	0.185	0.228±0.031
ND2	Duration of the 2nd note (sec)	0.243	0.253	0.267	0.233	0.249±0.013
ND3	Duration of the 3rd note (sec)	0.227	0.210	0.282	0.210	0.232±0.030
ND4	Duration of the 4th note (sec)	0.208	0.214	0.262	0.191	0.219±0.026
ND5	Duration of the 5th note (sec)	0.190	0.251	0.269	0.193	0.226±0.035
RT1	Rise time of the 1st note (sec)	0.187	0.206	0.176	0.163	0.183±0.016
RT2	Rise time of the 2nd note (sec)	0.138	0.098	0.145	0.115	0.124±0.019
RT3	Rise time of the 3rd note (sec)	0.163	0.047	0.225	0.081	0.129±0.070
RT4	Rise time of the 4th note (sec)	0.116	0.073	0.155	0.044	0.097±0.042
RT5	Rise time of the 5th note (sec)	0.114	0.111	0.108	0.064	0.099±0.020
FT1	Fall time of the 1st note (sec)	0.029	0.046	0.064	0.025	0.041±0.015
FT2	Fall time of the 2nd note (sec)	0.104	0.159	0.157	0.122	0.136±0.023
FT3	Fall time of the 3rd note (sec)	0.039	0.194	0.115	0.144	0.123±0.056
FT4	Fall time of the 4th note (sec)	0.088	0.145	0.101	0.160	0.124±0.030

FT5	Fall time of the 5th note (sec)	0.080	0.138	0.146	0.127	0.123±0.026
INI1	Inter-note interval between the 1st and 2nd notes (sec)	0.171	0.146	0.183	0.173	0.168±0.014
INI2	Inter-note interval between the 2nd and 3rd notes (sec)	0.188	0.144	0.200	0.169	0.175±0.021
INI3	Inter-note interval between the 3rd and 4th notes (sec)	0.231	0.174	0.216	0.180	0.200±0.024
INI4	Inter-note interval between the 4th and 5th notes (sec)	0.231	0.159	0.207	0.192	0.197±0.026
CD	Call duration (sec)	1.913	1.833	2.087	1.718	1.888±0.134

Table 6. The acoustic parameters measured for the calls of the five *Nidirana* species

Parameters	Definition	ND	NH	NO	NA	NL
FF1	Fundamental frequency of the 1st note (Hz)	460.425±37.423	667.475±37.282	703.300±63.474	592.100±35.699	947.550±475.421
FF2	Fundamental frequency of the 2nd note (Hz)	562.300±32.455	635.175±63.704	714.050±46.950	656.700±35.699	947.400±411.923
FF3	Fundamental frequency of the 3rd note (Hz)	607.050±34.171	635.400±82.397	725.775±48.579	667.475±48.154	958.125±445.818
FF4	Fundamental frequency of the 4th note (Hz)	607.050±34.171	709.075±92.194	736.525±53.316	678.225±35.737	979.650±426.686
FF5	Fundamental frequency of the 5th note (Hz)	607.300±34.143	699.775±82.699	747.300±35.185	678.225±35.737	958.150±423.421
DF1	Dominant frequency of the 1st note (Hz)	664.975±201.371	667.475±37.282	1083.775±233.454	1248.425±800.138	2012.900±921.648
DF2	Dominant frequency of the 2nd note (Hz)	702.100±242.871	1022.700±655.416	809.000±191.640	1226.975±703.787	2354.750±247.290
DF3	Dominant frequency of the 3rd note (Hz)	607.050±34.171	635.400±82.397	840.300±205.750	1280.725±773.864	2303.250±289.680
DF4	Dominant frequency of the 4th note (Hz)	607.050±34.171	999.550±524.408	840.300±205.750	1302.475±764.848	2351.000±188.481
DF5	Dominant frequency of the 5th note (Hz)	607.050±34.171	1055.000±639.030	870.000±214.291	1291.475±764.716	2260.250±236.886
ND1	Duration of the 1st note (sec)	0.210±0.024	0.094±0.027	0.043±0.002	0.360±0.131	0.228±0.031
ND2	Duration of the 2nd note (sec)	0.130±0.016	0.217±0.008	0.035±0.006	0.242±0.055	0.249±0.013
ND3	Duration of the 3rd note (sec)	0.126±0.013	0.224±0.009	0.032±0.005	0.213±0.064	0.232±0.030
ND4	Duration of the 4th note (sec)	0.118±0.014	0.222±0.003	0.029±0.005	0.185±0.048	0.219±0.026
ND5	Duration of the 5th note (sec)	0.118±0.005	0.220±0.007	0.028±0.006	0.176±0.035	0.226±0.035
RT1	Rise time of the 1st note (sec)	0.021±0.009	0.030±0.014	0.009±0.001	0.009±0.003	0.183±0.016
RT2	Rise time of the 2nd note (sec)	0.011±0.003	0.158±0.012	0.009±0.003	0.009±0.004	0.124±0.019
RT3	Rise time of the 3rd note (sec)	0.029±0.019	0.159±0.010	0.009±0.002	0.014±0.010	0.129±0.070
RT4	Rise time of the 4th note (sec)	0.015±0.009	0.159±0.008	0.006±0.001	0.014±0.010	0.097±0.042
RT5	Rise time of the 5th note (sec)	0.015±0.008	0.158±0.009	0.006±0.001	0.009±0.002	0.099±0.020
FT1	Fall time of the 1st note (sec)	0.180±0.031	0.059±0.009	0.026±0.004	0.345±0.130	0.041±0.015
FT2	Fall time of the 2nd note (sec)	0.116±0.017	0.059±0.003	0.027±0.006	0.219±0.062	0.136±0.023
FT3	Fall time of the 3rd note (sec)	0.107±0.017	0.058±0.005	0.026±0.004	0.206±0.070	0.123±0.056

FT4	Fall time of the 4th note (sec)	0.098±0.018	0.057±0.003	0.025±0.004	0.173±0.043	0.124±0.030
FT5	Fall time of the 5th note (sec)	0.102±0.008	0.058±0.006	0.024±0.004	0.173±0.022	0.123±0.026
INI1	Inter-note interval between the 1st and 2nd notes (sec)	0.159±0.005	0.141±0.022	0.049±0.006	0.196±0.028	0.168±0.014
INI2	Inter-note interval between the 2nd and 3rd notes (sec)	0.156±0.015	0.150±0.018	0.037±0.019	0.172±0.041	0.175±0.021
INI3	Inter-note interval between the 3rd and 4th notes (sec)	0.166±0.015	0.139±0.011	0.057±0.008	0.185±0.037	0.200±0.024
INI4	Inter-note interval between the 4th and 5th notes (sec)	0.179±0.021	0.150±0.016	0.056±0.005	0.206±0.037	0.197±0.026
CD	Call duration (sec)	1.373±0.041	1.560±0.051	1.563±0.032	1.941±0.307	1.888±0.134

Note: For a given *Nidirana* species, the values were averaged across the four exemplars for each acoustic parameter (mean ± SD). ND, call of *N. daunchina*; NH, call of *N. hainanensis*; NO, call of *N. okinavana*; NA, call of *N. adenopleura*; NL, call of *N. lini*.

Table 7. Localities, voucher information and GenBank numbers for all samples used in the present study

Species	Locality	Coordinate	GenBank accession no. (12S/16S r-RNA)
N. daunchina	Mt. Emei, Sichuan Province	29.35°N, 103.17°E	KF185029, KF185065
N. adenopleura	Kuankuoshui, Guizhou Province	28.13° N and 107.09° E	NC_018771, NC_018771
N. hainanensis	Mt. Diaoluo, Hainan Province	18.44° N and 109.52° E	MF807899, MF807821
N. okinavana	Iriomote Island, Okinawa, Japan	Not given	NC022872, NC022872
N. lini	Jiangcheng, Yunnan Province	22.40° N and 101.52° E	MF807898, KF185066
O. exiliversabilis	Wuyishan, Fujian	Not given	KF185020, KF185056
O. tormotus	Huangshan, Anhui	Not given	EF453739, EF453754
O. margaretae	China	Not given	NC_024603.1, NC_024603.1
