# Supplementary material for: Neural activities in music frogs reveal call variations and phylogenetic relationships within the genus Nidirana
Source: Commun Biol. 2022 Jun 6;5:550. doi: 10.1038/s42003-022-03504-8 (PMC9170687; doi:10.1038/s42003-022-03504-8)
Supplement: Supplementary file 3 — Description of Additional Supplementary Files [file 42003_2022_3504_MOESM3_ESM.pdf]

Description of Additional Supplementary Files

File name: Supplementary Data 1
Description: The source data behind Figure 1a in the paper

File name: Supplementary Data 2
Description: The source data behind Figure 1b in the paper

File name: Supplementary Data 3
Description: The source data behind Figure 2 in the paper
