# Supplementary material for: Neural activities in music frogs reveal call variations and phylogenetic relationships within the genus Nidirana
Source: Commun Biol. 2022 Jun 6;5:550. doi: 10.1038/s42003-022-03504-8 (PMC9170687; doi:10.1038/s42003-022-03504-8)
Supplement: Supplementary file 7 — Reporting Summary [file 42003_2022_3504_MOESM7_ESM.pdf]

nature research

Corresponding author(s): Guangzhan Fang

Last updated by author(s): Apr 26, 2022

Reporting Summary

Nature Research wishes to improve the reproducibility of the work that we publish. This form provides structure for consistency and transparency in reporting. For further information on Nature Research policies, see our Editorial Policies and the Editorial Policy Checklist.

Statistics

For all statistical analyses, confirm that the following items are present in the figure legend, table legend, main text, or Methods section.

n/a	Confirmed	
☐	☒	The exact sample size (*n*) for each experimental group/condition, given as a discrete number and unit of measurement
☐	☒	A statement on whether measurements were taken from distinct samples or whether the same sample was measured repeatedly
☐	☒	The statistical test(s) used AND whether they are one- or two-sided<br>*Only common tests should be described solely by name; describe more complex techniques in the Methods section.*
☒	☐	A description of all covariates tested
☐	☒	A description of any assumptions or corrections, such as tests of normality and adjustment for multiple comparisons
☐	☒	A full description of the statistical parameters including central tendency (e.g. means) or other basic estimates (e.g. regression coefficient) AND variation (e.g. standard deviation) or associated estimates of uncertainty (e.g. confidence intervals)
☐	☒	For null hypothesis testing, the test statistic (e.g. *F*, *t*, *r*) with confidence intervals, effect sizes, degrees of freedom and *P* value noted<br>*Give P values as exact values whenever suitable.*
☐	☒	For Bayesian analysis, information on the choice of priors and Markov chain Monte Carlo settings
☐	☒	For hierarchical and complex designs, identification of the appropriate level for tests and full reporting of outcomes
☐	☒	Estimates of effect sizes (e.g. Cohen's *d*, Pearson's *r*), indicating how they were calculated

Our web collection on statistics for biologists contains articles on many of the points above.

Software and code

Policy information about availability of computer code

Data collection	No software was used.
Data analysis	We used Adobe Audition 3.0 software (San Jose, California, USA) and Praat software (version 6.1.13) to analyze acoustic parameters. We used EEGLAB toolbox to analyze ERPs. We used MEGA X, MrBayes version 3.1.2 software, and FigTree v1.3.1 for phylogenetic analyses.

For manuscripts utilizing custom algorithms or software that are central to the research but not yet described in published literature, software must be made available to editors and reviewers. We strongly encourage code deposition in a community repository (e.g. GitHub). See the Nature Research guidelines for submitting code & software for further information.

Data

Policy information about availability of data

All manuscripts must include a data availability statement. This statement should provide the following information, where applicable:

- Accession codes, unique identifiers, or web links for publicly available datasets
- A list of figures that have associated raw data
- A description of any restrictions on data availability

The dataset generated and analyzed in the current study are available at http://dx.doi.org/ 10.17632/yfnk4zt2hk.1

Field-specific reporting

Please select the one below that is the best fit for your research. If you are not sure, read the appropriate sections before making your selection.

☒ Life sciences ☐ Behavioural & social sciences ☐ Ecological, evolutionary & environmental sciences

For a reference copy of the document with all sections, see nature.com/documents/nr-reporting-summary-flat.pdf

Life sciences study design

All studies must disclose on these points even when the disclosure is negative.

Sample size	We did not use any methods to predetermine sample size. Many studies on EEG in animals used ten subjects. In our study, 16 frogs were used because of controlling negative effects of pseudoreplication.
Data exclusions	No data were excluded from the analyses.
Replication	Because pseudoreplication may affect the conclusions of statistical analyses in ecological, animal behavior and neuroscience studies, its possible effects were controlled by using multiple stimulus exemplars (i.e. four stimulus sets) in the present study. There were no significant main effects for the factor “stimulus set”, consistent with the idea that the four stimulus sets were not significantly different at eliciting responses from the subjects.
Randomization	All playback orders were randomized. And for the deviant stimuli, randomization was constrained to prevent successive presentation of three or more stimuli from the same acoustic category.
Blinding	The investigators were blinded to group allocation during data collection and data analysis.

Reporting for specific materials, systems and methods

We require information from authors about some types of materials, experimental systems and methods used in many studies. Here, indicate whether each material, system or method listed is relevant to your study. If you are not sure if a list item applies to your research, read the appropriate section before selecting a response.

Materials & experimental systems

n/a	Involved in the study	
☒	☐	Antibodies
☒	☐	Eukaryotic cell lines
☒	☐	Palaeontology and archaeology
☐	☒	Animals and other organisms
☒	☐	Human research participants
☒	☐	Clinical data
☒	☐	Dual use research of concern

Methods

n/a	Involved in the study	
☒	☐	ChIP-seq
☒	☐	Flow cytometry
☒	☐	MRI-based neuroimaging

Animals and other organisms

Policy information about studies involving animals; ARRIVE guidelines recommended for reporting animal research

Laboratory animals	The study did not involve laboratory animals.
Wild animals	Sixteen adult Emei music frogs (eight females and eight males) (N. daunchina) were captured from the Emei mountain area (29.35°N, 103.17°E, elevation of 1315 m above sea level) of Sichuan, China, during the breeding season, using a butterfly net. After all experiments were completed, the subjects were euthanized by overdose of MS-222 solution for confirming the electrode locations.
Field-collected samples	The subjects were separated by sex in opaque plastic tanks (45 × 35 cm, and 30 cm deep) containing about 2 cm depth of water with about 5 cm of mud around the bottom walls. The tanks were placed in a room under controlled temperature conditions (23 ± 1°C) and relative humidity (70 - 80%) with a 12:12 light-dark cycle (lights on at 08:00) using a fluorescent lamp. The animals were fed fresh live crickets (bought from a pet food shop) every three days.
Ethics oversight	All studies were conducted under approval of the Animal Care and Use Committee of Chengdu Institute of Biology, Chinese Academy of Sciences (permit number: 20191203).

Note that full information on the approval of the study protocol must also be provided in the manuscript.
